# The Societal Burden of Heart Failure With Preserved or Mid-Range Ejection Fraction

**DOI:** 10.1016/j.jacadv.2024.101025

**Published:** 2024-07-24

**Authors:** William S. Weintraub, Maria Alva

**Affiliations:** a Director of Population Health Research, MedStar Health Research Institute, Professor of Medicine, Georgetown University, Washington, DC, USA; b Assistant Professor of Health Policy and Management, Georgetown University, Washington, DC, USA

**Keywords:** heart failure costs, heart failure mortality, heart failure quality of life

**Figure:**
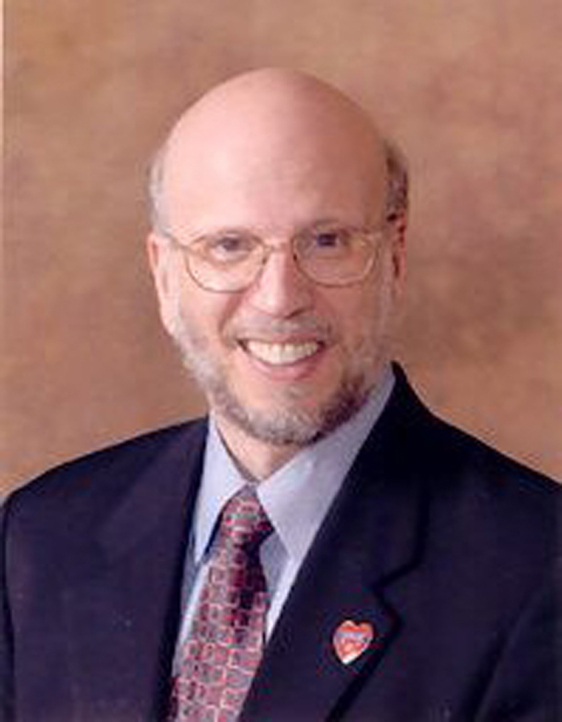

Heart failure remains a major health care issue with high prevalence, symptom burden, mortality, and costs. Historically, there has been greater concern with heart failure with reduced ejection fraction (HFrEF). However, in recent years, the frequency and burden of heart failure with preserved ejection fraction (HFpEF) or heart failure with mid-range ejection fraction (HFmrEF) is being increasingly recognized.^1^ Much of the outcomes data available comes from clinical trials on patients with HFrEF. While of great importance, such patients may not represent the full spectrum of patients with disorders encapsulated as HFpEF/HFmrEF, which are typically older and more commonly female.^2^

Recent trials (post 2019) have started to investigate treatments in HFpEF and HFmrEF patients specifically. The median follow-up in these trials is <3 years. In this issue of *JACC: Advances*, Sun et al^3^ with the research led by Sean Sullivan, studied the long-term costs and outcomes of treatment in this population based on the EMPEROR Preserved, PARAGON-HF, and DELIVER trials, which respectively compared empagliflozin to placebo, sacubitril-valsartan to valsartan, and dapagliflozin to placebo.^4^, ^5^, ^6^ The authors developed a Markov model where patients start in a state with stable, symptomatic heart failure and ejection fraction ≥40%. Patients were at risk of heart failure hospitalizations, treatment discontinuation, hyperkalemia, renal endpoints, or death. Life years and costs were discounted 3% per year, with 5 and 10 year time horizons. Event rates were extrapolated beyond the trial period from in-trial event rates. In the trials, the weighted average baseline age was 72.0 years and 53.6% were male. It was estimated that 20% of patients were treated with a sodium-glucose cotransporter-2 (SGLT2) inhibitor, based on real world data from patients with HFrEF as there is no published evidence currently on the uptake of SGLT2 inhibitors in the HFpEF/HFmrEF populations. Costs in 2023 USD were estimated from multiple sources in the literature.^7^, ^8^, ^9^ The estimated 10-year incidence of heart failure hospitalizations was 0.53 per patient. Overall, 37% had 1 or more heart failure hospitalizations and 26% suffered cardiovascular death. Estimated survival was 6.1 years. Total cost of care was $123,900, with $49,900 attributable to stable heart failure costs, $33,600 due to noncardiovascular, and $20,800 due to cardiovascular death costs. Costs were sensitive to the proportion of patients treated with SGLT2 inhibitors, cost of stable heart failure care, and cost of noncardiovascular and cardiovascular death.

The authors acknowledge a number of limitations, including uncertainty as to event rates, uptake of SGLT2 inhibitors, and costs. There is also uncertainty as to the adherence to all heart failure medications. Furthermore, the trial data may not be generalizable to the larger community of patients with HFpEF/HFmrEF, who may have greater comorbidity than the trial participants. In addition, there are costs that were not measured, including non-heart failure hospitalizations, outpatient care, rehabilitation, non-heart failure drug costs, family, and other home care providers and, for younger patients, lost wages. The limitations of this study, which would be noted in most studies of health care costs, make it difficult to estimate the true costs of care for patients suffering HFpEF/HFmrEF. Almost certainly, the true costs will be higher than noted in this study, possibly substantially higher.

What does the care of patients with HFpEF/HRmrEF really cost? The answer is that in a mixed product environment, it is impossible to know what anything really costs. This is in part because of the cross-subsidization of costs across services. It is also due to the problem of attribution when resources are shared.^10^ When should we attribute a cost to HFpEF/HRmrEF as opposed to another related health care concern? For instance, patients with HFpEF/HRmrEF are at risk of atrial fibrillation. The care of patients with atrial fibrillation can also be expensive with the need for ablation and other procedures. If HFpEF/HRmrEF is in the causal path leading to atrial fibrillation, then some of the cost of care for atrial fibrillation could be attributed to HFpEF/HRmrEF. In principle, this could be done by establishing the attributable risk of HFpEF/HRmrEF to the subsequent development of atrial fibrillation.^11^ However, there will be conditions in which HFpEF/HFmrEF does not necessarily contribute to attributable risk but may increase total cost. For instance, the care of patients requiring abdominal surgery may be increased in patients with HFpEF/HFmrEF. In principle, the contribution of HFpEF/HFmrEF to costs could also be assessed by regression analysis. However, such approaches have rarely been used because regression analysis can identify associations between a condition and costs but cannot definitely establish attribution. Besides the challenge to disentangle the specific contribution of a condition to overall costs, health care utilization affects illness severity which in turn influences costs.

If we go beyond the individual patient, what is the societal economic burden of HFpEF/HFmrEF? To assess this, we would need to know both the prevalence of HFpEF/HFmrEF and at least the average cost. The 2021 American Heart Association Statistical update estimates the prevalence of heart failure in the United States at 6 million people or 1.8% of the population.^12^ If half of these people had HFpEF/HFmrEF, this would be 3 million people. The prevalence increases dramatically with age. If the care of these patients were on the order of $20,000 a year, this would be in the range of $60 billion a year, with costs increasing over time as the population ages. This estimate aligns with the context of total healthcare costs exceeding $3 trillion in the United States alone and whereby, HFpEF/HRmrEF would represent 2% of these costs.^13^ Heart failure is a major problem worldwide, with an overall prevalence estimate of 67 million people, with widely varying estimates of costs between countries, even between wealthier nations.^14^

What can be done about this terrible problem? Newer agents, including SGLT2 inhibitors, may yet be shown to reduce hospitalizations and associated costs in patients with HFpEF/HFmrEF. However, such agents have not been shown to reduce total costs, and incremental cost effectiveness ratios are likely to remain above $50,000 a year. Prevention is the most important approach. The most important causes of heart failure remain ischemic heart disease and hypertension. Ischemic heart disease is preventable by risk factor control. Hypertension is treatable, with new therapies being developed in recent years. However, recognition and treatment of hypertension remain inadequate and a major problem in both the developed and developing worlds.

In summary, it is clear that heart failure in general and HFpEF/HFmrEF in particular remains a major public health issue worldwide with considerable morbidity, loss of life, and associated health care costs. Care for these patients will continue to pose a significant societal burden. Sun et al^3^ have pointed out the very real economic burden of providing this care and carefully note many of the limitations. Continuing research should focus on the effects of HFpEF/HFmrEF on health status and quality of life, long-term outcome, and potential benefits of associated therapy as well as resource consumption and societal burden. Furthermore, such research is necessary in the developing world as well as in wealthier countries. At the same time, we must redouble our efforts to mitigate individual and societal modifiable risk factors (over-promotion and consumption of high calorie processed foods and sodium, lack of regular exercise, excessive alcohol intake, tobacco consumption, chronic stress, lack of reliable primary care, and preventive healthcare access and associated lack of health care equity) to prevent the very real detrimental effects of this pervasive syndrome.

## Funding support and author disclosures

Drs Weintraub and Alva have received a grant from Lexicon Pharmaceuticals.
